# Real-World Acceptance of COVID-19 Vaccines among Healthcare Workers in Perinatal Medicine in China

**DOI:** 10.3390/vaccines9070704

**Published:** 2021-06-27

**Authors:** Biyun Xu, Xuelian Gao, Xinyue Zhang, Yali Hu, Huixia Yang, Yi-Hua Zhou

**Affiliations:** 1 Departments of Laboratory Medicine and Biomedicine Statistics, Nanjing Drum Tower Hospital, Nanjing University Medical School, Nanjing 210008, China; biyunxu@163.com; 2 Department of Obstetrics and Gynecology, Peking University First Hospital, Beijing 100034, China; zhwc_gaoxuelian@163.com (X.G.); zhwc_zhangxinyue@163.com (X.Z.); 3 Department of Obstetrics and Gynecology, Nanjing Drum Tower Hospital, Nanjing University Medical School, Nanjing 210008, China; dtylhu@126.com; 4 Department of Infectious Diseases, Nanjing Drum Tower Hospital, Nanjing University Medical School, Nanjing 210008, China

**Keywords:** coronavirus disease 2019 (COVID-19), vaccine acceptance, healthcare workers, China

## Abstract

Surveys showed that vaccine hesitancy may influence the acceptance of COVID-19 vaccines in healthcare workers (HCWs) and the general population. Currently, the actual acceptance of COVID-19 vaccination in HCWs has rarely been reported. In the present survey, we investigated the real-world acceptance of COVID-19 vaccination in HCWs in perinatal medicine during the first three-month period of vaccination in China and to identify the main reason for the decline of vaccination. HCWs (1087) who participated in a Chinese national symposium on perinatal medicine during 16–18 April 2021 were invited to answer a 27-question questionnaire online. A total of 1051 HCWs completed the questionnaire. Of them, 86.2% (906/1051) accepted the COVID-19 vaccination and 13.8% (145/1051) declined the vaccination. Because of the vaccine hesitancy, one-fourth of the vaccinated participants did not accept the vaccination until consulted with others or requested by employers. The main reason for the decline of vaccination in 145 unvaccinated HCWs was the concern about vaccine safety. The results indicate that vaccination request by employers may promote vaccine acceptance. More convincing data on the safety of COVID-19 vaccines appears to be important to increase the acceptance of vaccination.

## 1. Introduction

Coronavirus disease 2019 (COVID-19), caused by severe acute respiratory syndrome coronavirus 2 (SARS-CoV-2), is still overwhelmingly spreading in the world. As of 8 June 2021, a total of 172.64 million COVID-19 patients were confirmed worldwide with 3.42 million deaths [1]. The successful experience in the eradication of smallpox and in the elimination of poliovirus by universal vaccinations provides good examples for controlling the COVID-19 pandemic by universal vaccination against COVID-19. Fortunately, animal experiments and clinical trials demonstrated that several types of COVID-19 vaccines are effective in preventing SARS-CoV-2 infection [2,3,4,5,6,7]. Currently, various COVID-19 vaccines are being used throughout the world.

While the development of effective COVID-19 vaccines is the first step to contain the pandemic of COVID-19, another critical step is to achieve herd immunity by the widespread use of COVID-19 vaccines in the general population. However, vaccine hesitancy, a phenomenon in which some individuals delay or decline to take one or more vaccines when vaccination services are available and accessible, is one of main obstacles to control pandemics of infectious diseases [8,9]. Recently, numerous surveys reported the intention to receive COVID-19 vaccines in the general population as well as in healthcare workers (HCWs) [10,11]. The results showed that the potential acceptance of a COVID-19 vaccine in the general population was different, ranging from 23.6% in Kuwait and 28.4% in Jordan [10], 62.1% in Japan [12], 69% in USA [13], to 90% in England [14] and in mainland China [15]. Moreover, the longitudinal surveys showed that the willingness to get COVID-19 vaccines had a decreasing trend in the USA [16]. Surveys among HCWs showed that COVID-19 vaccine willingness rates varied considerably in different countries or regions, ranging from 23.4% in Taiwan [17], 34.9% in Republic of Cyprus [18], 50.5% in the Kingdom of Saudi Arabia [19], 40.0–63% in Hong Kong [20,21], 46.9–63.7% in USA [22,23,24,25], 51.1–64.4% in Greece [18,26], 76.9% in France [27], to 79.1% in China [11]. Overall, the COVID-19 vaccination intention in HCWs appears to be somewhat lower than in general populations. While the determinants of acceptance or decline of the COVID-19 vaccine appear to be complex, some psychological structures are shown to be associated with vaccine hesitancy, such as personality traits neuroticism, paranoid or conspiracy beliefs, distrust of government officials, scientists, and healthcare professionals in vaccinology, immunology, and infectious diseases [28,29,30].

As of 30 December 2020, the China Health Authority issued the first licensed COVID-19 vaccine, composed of inactivated SARS-CoV-2, for emergency use in adult populations (at 18–60 years age) at risk for infection. The full vaccination requires two injections at an interval of two to four weeks. China has taken a policy to vaccinate all staff employed in hospitals as well as other populations at high risk for infection of SARS-CoV-2 during the initial period of the vaccination campaign. It was scheduled to complete the COVID-19 vaccination in all HCWs between 1 January and 31 March 2021. Although vaccination intention may determine whether or not to take a COVID-19 vaccine, expression of willingness to take COVID-19 vaccine may be different from the actual acceptance of the vaccine. A person who initially intended to receive the COVID-19 vaccine may decline to accept vaccination when the vaccine is available, and vice versa.

Because pregnant women are at increased risk for SARS-CoV-2 infection and development of more severe illness [31,32], although the initial observation did not reveal the severe adverse influence of COVID-19 on the outcomes of pregnancy [33], HCWs in obstetrics and gynecology and neonatology are at high risk for occupational SARS-CoV-2 exposure and transmission. Therefore, vaccination against COVID-19 in these HCWs appears to be more important. To date, no study on the actual acceptance of the COVID-19 vaccine in HCWs in obstetrics and gynecology and neonatology was reported. The current study aimed to survey the actual acceptance of a COVID-19 vaccine in HCWs in perinatal medicine in China.

## 2. Materials and Methods

### 2.1. Study Design

This was a cross-sectional survey about the actual acceptance of the COVID-19 vaccine among participants in a nation-wide symposium on the perinatal medicine held in Taiyuan city, Shanxi Province, China, 16–18 April 2021. The survey was conducted by an online platform, started one week before the symposium (9 April 2021) and completed three days after the end of symposium (21 April 2021). All participants who pre-registered or onsite registered for the symposium were invited to complete the questionnaire form. The inclusion criteria for HCWs were (1) an HCW in perinatal medicine who attended the symposium; (2) at the age of 18–60 years, because the indication for COVID-19 vaccination was 18–60 years old in the first period of vaccination campaign in China; and (3) willing to participate in the study. The participants were informed at the beginning of the questionnaire form before they started to answer the survey items. This study was approved by Ethics Committee of the Nanjing Drum Tower Hospital (2021-138-01) and the Peking University First Hospital (PUFH-21-196).

### 2.2. Survey Content

We prepared 27 questions, including 15 questions in 3 sections (basic demographic information, institutions information, knowledge of and attitudes to COVID-19 vaccine) for all participants, one question of whether acceptance of the vaccine, and 10 questions for those who were vaccinated, and one question for those who declined the vaccination. The detailed questions in Chinese and English are presented in Questionnaire-Chinese and English in Supplementary file 1.

### 2.3. Statistical Analysis

Categorical variables were represented as number and percentage. The rates or proportions of different group were compared by the chi-squared test or the chi-squared test with Yates’ correction for continuity. The Mann–Whitney U test was used to test the difference between two groups on an ordinal categorical variable. Variables that were significant at *p* < 0.1 in univariate analyses were then included in a multiple logistic regression. *p* < 0.05 was considered statistically significant. All analyses were performed using statistical analysis system software, Version 9.4 (SAS Institute Inc., Cary, NC, USA).

## 3. Results

### 3.1. Participant Characteristics

In total, 1087 HCWs participated in the survey and 1051 (96.7%) subjects who completed the survey were included in the analyses. The excluded participants were those who provided incomplete responses (*n* = 36). Overall, 906 (86.2%) subjects received a COVID-19 vaccine (all vaccinated inactivated SARS-CoV-2 vaccine except two subjects vaccinated with recombinant adenovirus vaccine) and 145 (13.8%) others did not. Of the 906 vaccinated participants, two received the recombinant adenovirus vaccine and required no second dose, 644 completed the second vaccine dose, and 260 others had not yet received the second dose because the intervals were less than two weeks. Participants’ basic information of demographic characteristics, educational levels, and professional roles among those who received the COVID-19 vaccine and those who did not receive the vaccine is summarized in Table 1. Whether or not acceptance of COVID-19 vaccination was not associated with genders, nationalities, religions, educational levels, roles in hospitals, or departments, but was associated with ages, titles, and hospital levels. Subjects at the age of 18–30 years, and those with primary titles showed lower acceptance, and those employed in high level hospitals had a low acceptance rate (Table 1).

### 3.2. Knowledge of and Attitude to COVID-19 Vaccine

The first approved COVID-19 vaccine in China is composed of inactivated SARS-CoV-2. The vaccine experts and professional healthcare providers repeatedly explained the safety and efficacy of the COVID-19 vaccine by means of various news media to the public in China just before and during the vaccination campaign. Table 2 shows that basic knowledge of and the attitudes to the COVID-19 vaccine in the participants who accepted the COVID-19 vaccine and those who did not receive the vaccine. The results showed that HCWs who declined the COVID-19 vaccine were more worried about vaccine safety.

### 3.3. Factors Associated with Vaccine Acceptance

Table 3 shows the results of binary logistic regression analysis on the factors associated with the acceptance of the COVID-19 vaccine. Those who had junior high professional title, were working in middle level hospitals, or had no/little worry about the vaccine safety were more likely to accept the vaccine.

### 3.4. Reasons for Acceptance or Decline of the COVID-19 Vaccine

Table 4 shows the reasons for the acceptance of COVID-19 vaccination in 906 vaccinated participants. Three-fourths of the vaccinated participants took the vaccine based on their own decision without a specific reason. The remaining one-fourth had hesitancy before they accepted the vaccination. The detailed reasons for declining COVID-19 vaccination are presented in Table 5. Those who worried about the vaccine safety and were in pregnancy and lactation accounted for 72.4% (105/145).

### 3.5. Self-Reported Adverse Events in 906 Vaccinated Participants

Of the 906 vaccinated participants, 594 (65.6%) reported no adverse events at all and 312 (34.4%) others reported one or more adverse events within two weeks after the injection of first dose vaccine. Of those with adverse events, 74.0% (231/312) only had local ache and/or swelling on the injection site. Overall, 93.3% (291), 6.7% (21), and 0% (0) of these 312 vaccinated participants had very mild/mild, moderate, and severe adverse events, respectively. The detailed adverse events in the 312 vaccinated participants are presented in Table S1 in the Supplementary File. Of the 644 vaccinated participants who completed the second vaccine dose, 481 (74.7%) reported no any adverse events at all, and 163 (25.3%) others reported one or more adverse events. The local reaction on injection site accounted for 81.0% (132/163), and the vast majority (96.3%, 157/163) were mild and none had severe adverse events. Table S2 in the Supplementary File presents the detailed adverse events in these 163 vaccinated participants.

## 4. Discussion

In this study, we surveyed the actual acceptance of the COVID-19 vaccine in HCWs in perinatal medicine, mostly in obstetrics, gynecology, and neonatology, during the first period (1 January to 31 March 2021) of the vaccination campaign against COVID-19 in China, and found that 86.2% (906/1051) of HCWs already received the COVID-19 vaccine and 13.8% (145/1051) declined the vaccination. Vaccination request by employers promoted the acceptance. The main reason for the decline of the COVID-19 vaccine is the concern about the vaccine safety.

Surveys on the COVID-19 vaccination intention among HCWs showed that the vaccine willingness rates varied considerably in different countries or regions, ranging from 23.4 to 79.1% [11,17,18,19,20,21,22,23,24,25,26,27]. During the first month of the vaccination program in the US, the median first-dose COVID-19 vaccination coverage among skilled nursing facility staff was just 37.5% [34]. In the present survey, the real-world acceptance of COVID-19 vaccination reached to 86.2% in HCWs in perinatal medicine, higher than the reported vaccination willingness rate in China [11] and in other countries or regions [17,18,19,20,21,22,23,24,25,26,27]. The relatively higher acceptance of COVID-19 vaccination appeared to be associated to education and vaccination request by the employers, because 23.4% (212) and 2.2% (20) of the 906 vaccinated participants were hesitant and not willing to be vaccinated respectively before their acceptance of vaccination (Table 4). Thus, various vaccination educational actions as well as the vaccination request by the employers may promote HCWs to take the vaccine. This is practically meaningful to increase vaccine acceptance in real-world practice.

The main detailed reasons for the decline of vaccination among those 145 unvaccinated subjects included the concern about the vaccine safety, decline by vaccination staff, pregnancy or lactation (Table 5). Studies demonstrated that the inactivated COVID-19 vaccine is safe [2,4,35,36], which is also observed in the present survey (Tables S1 and S2). Actually, the chronic diseases under controlled conditions, such as hypertension, diabetes mellitus, supplement of thyroid hormone and others, are not the contradiction of vaccination [37,38]. Decline by vaccination staff is also related to the safety of vaccine. Thus, more safety data of the COVID-19 vaccine in subjects with these conditions are required.

Vaccination in women with lactation is generally safe [39], except for yellow fever vaccine, which is an attenuated live vaccine that may cause yellow fever infection in infant because of the secretion of live virus in breastmilk [40]. Vaccination with inactivated vaccines in pregnant women has no additional adverse events, compared to vaccination in general populations [39]. Because inactivated COVID-19 vaccine, as well as the mRNA vaccine, does not contain live microbes, the vaccines should be in theory safe in pregnant women. Recent studies showed that the COVID-19 vaccine does not cause additional adverse effects in pregnant or lactating women [41,42]. However, surveys showed that only 28.2% of pregnant women in Italy were willing to get vaccinated [43]. This finding is in agreement with the observation that the scientific-sounding misinformation appears to be associated with the decline of the COVID-19 vaccine [44]. Thus, more educational campaigns and safety data of the COVID-19 vaccine in pregnant women and women with lactation is required.

There are several limitations in the present study. The first is that most participants in the survey were from high levels of hospitals in cities, because HCWs in communities or towns seldom attend the national symposium. The second is that we just investigated the COVID-19 vaccine acceptance during the first period of the vaccination campaign. Since 1 April 2021, the universal vaccination campaign against COVID-19 has started in general populations, including subpopulations at-risk for infection in China. A proportion of HCWs who did not receive the vaccine in the first period of vaccination campaign may likely receive the vaccine in the coming days. The third is that HCWs were from China, located in Asia. The surveys on COVID-19 vaccination attention showed that Asians are much more likely to take the COVID-19 vaccine than Europeans [10]. Thus, more surveys are required to estimate the actual acceptance of COVID-19 vaccination in Western countries.

## 5. Conclusions

This survey shows that 86.2% of HCWs in perinatal medicine in China were already vaccinated against COVID-19 in the first three-month period of vaccination. Vaccination education and request by employers may increase the vaccine acceptance. The main reason for declining the COVID-19 vaccine appears to be the concern about vaccine safety, which highlights the urgent requirement of convincing evidence on the safety of the vaccine.

## Figures and Tables

**Table 1:** Sociodemographic characteristics of participants.

Variable	Total, *n* = 1051 (%)	Vaccinated, *n* = 906 (%)	Unvaccinated, *n* = 145 (%)	Statistic ^1^	*p*
Gender				χ^2^ = 0.040	0.842
Male	111 (10.6)	95 (10.5)	16 (11.0)		
Female	940 (89.4)	811 (89.5)	129 (89.0)		
Nationality				χ^2^ = 0.636	0.425
Han	1001 (95.2)	861 (95.0)	140 (96.6)		
Minority	50 (4.8)	45 (5.0)	5 (3.4)		
Religion				χ^2corr^ = 0.000	1.000
Yes	15 (1.4)	13 (1.4)	2 (1.4)		
No	1036 (98.6)	893 (98.6)	143 (98.6)		
Age (years)				z = 2.007	0.045
18–30	204 (19.4)	165 (18.2)	39 (26.9)		
31–40	378 (36.0)	323 (35.7)	55 (37.9)		
41–50	279 (26.5)	259 (28.6)	20 (13.8)		
51–60	190 (18.1)	159 (17.5)	31 (21.4)		
Education				z = 0.073	0.942
Doctorial degree	92 (8.7)	80 (8.8)	12 (8.3)		
Master	277 (26.4)	239 (26.4)	38 (26.2)		
University (Bachelor)	603 (57.4)	518 (57.2)	85 (58.6)		
Below University	79 (7.5)	69 (7.6)	10 (6.9)		
Departments				χ^2^ = 4.339	0.114
Obs/Gyn	792 (75.3)	687 (75.8)	105 (72.4)		
Pediatric	150 (14.3)	132 (14.6)	18 (12.4)		
Others	109 (10.4)	87 (9.6)	22 (15.2)		
Roles				χ^2^ = 6.261	0.100
Physician	670 (63.8)	582 (64.2)	88 (60.7)		
Nurse/midwife	293 (27.9)	254 (28.0)	39 (26.9)		
Laboratory technician	56 (5.3)	42 (4.6)	14 (9.7)		
Others	32 (3.0)	28 (3.1)	4 (2.8)		
Professional title				z = 2.919	0.004
High (senior)	160 (15.2)	135 (14.9)	25 (17.2)		
High (junior)	196 (18.7)	186 (20.5)	10 (6.9)		
Middle	352 (33.5)	307 (33.9)	45 (31.0)		
Primary	343 (32.6)	278 (30.7)	65 (44.8)		
Hospital level				z = 3.595	<0.0001
High	838 (79.7)	706 (77.9)	132 (91.0)		
Middle	177 (16.9)	167 (18.4)	10 (6.9)		
Low	36 (3.4)	33 (3.6)	3 (2.1)		
University hospital				χ^2^ = 0.050	0.824
Yes	629 (59.8)	541 (59.7)	88 (60.7)		
No	422 (40.2)	365 (40.3)	57 (39.3)		

^1^ The chi-squared test (χ^2^) was used to compare the group differences in gender, nationality, departments, roles, and university hospital. The chi-squared test with Yates’ correction for continuity (χ^2corr^) was used to compare the group differences in religion because an expected frequency is greater than 1 and smaller than 5. The Mann-Whitney U test (z) was used to compare the group differences in age, education, professional title, and hospital level.

**Table 2:** Participants’ knowledge of and attitudes to COVID-19 vaccine.

Variable	Total, *n* = 1051 (%)	Vaccinated, *n* = 906 (%)	Unvaccinated, *n* = 145 (%)	Statistic ^1^	*p*
Vaccine type used in China				χ^2^ = 1.023	0.600
Correct answer (%)	781 (74.3)	677 (74.7)	104 (71.7)		
Wrong answer (%)	157 (14.9)	135 (14.9)	22 (15.2)		
Unknown (%)	113 (10.8)	94 (10.4)	19 (13.1)		
Knowledge source					
News media	844 (80.3)	721 (79.6)	123 (84.8)	χ^2^ = 2.176	0.140
Academic journals	450 (42.8)	394 (43.5)	56 (38.6)	χ^2^ = 1.209	0.271
From collegues	439 (41.8)	371 (40.9)	68 (46.9)	χ^2^ = 1.818	0.178
From experts	579 (55.1)	500 (55.2)	79 (54.5)	χ^2^ = 0.025	0.874
From lecture in hospital	632 (60.1)	548 (60.5)	84 (57.9)	χ^2^ = 0.340	0.560
From lecture in symposium	255 (24.2)	215 (23.7)	40 (27.6)	χ^2^ = 1.011	0.315
Almost unknown	28 (2.7)	23 (2.5)	2 (1.4)	χ^2corr^ = 0.310	0.577
Safety of a COVID-19 vaccine				z = 2.233	0.026
Not worry/Little worry	714 (67.9)	626 (69.1)	88 (60.7)		
Somewhat worry	179 (17.0)	153 (16.9)	26 (17.9)		
Worry/very worry	158 (15.1)	127 (14.0)	31 (21.4)		
Effectiveness of a COVID-19 vaccine				z = 1.529	0.126
High	756 (71.9)	659 (72.7)	97 (66.9)		
Medium	200 (19.1)	169 (18.7)	31 (21.4)		
Low	81 (7.7)	67 (7.4)	14 (9.7)		
Not at all	14 (1.3)	11 (1.2)	3 (2.1)		

^1^ The chi-squared test (χ^2^) was used to compare the group differences in vaccine type used in China, knowledge source from news media, academic journals, colleagues, experts, lecture in employment hospital, and lecture in symposium. The chi-squared test with Yates’ correction for continuity (χ^2corr^) was used to compare the group differences in almost unknown knowledge because an expected frequency is greater than 1 and smaller than 5. The Mann–Whitney U test (z) was used to compare the group differences in safety and effectiveness of a COVID-19 vaccine.

**Table 3:** Factors associated with COVID-19 vaccine acceptance: Binary logistic regression ^1^.

Variables	β	SE	P	OR	95%CI	
Age (years)						
18–30	-	-	0.133	1.000	-	-
31–40	−0.008	0.279	0.976	0.992	0.574	1.714
41–50	0.252	0.407	0.535	1.287	0.580	2.858
51–60	−0.579	0.448	0.196	0.561	0.233	1.349
Professional title						
Primary	-	-	0.022	1.000	-	-
Middle	0.388	0.279	0.165	1.474	0.852	2.549
High (junior)	1.378	0.447	0.002	3.968	1.653	9.529
High (senior)	0.609	0.440	0.166	1.839	0.776	4.358
Hospital level						
High	-	-	0.007	1.000	-	-
Middle	1.040	0.343	0.002	2.828	1.444	5.540
Low	0.644	0.617	0.297	1.903	0.568	6.381
Safety of a COVID-19 vaccine						
Very worry/Worry	-	-	0.105	1.000	-	-
Somewhat worry	0.319	0.299	0.287	1.375	0.765	2.472
Not worry/Little worry	0.499	0.237	0.035	1.647	1.036	2.619

^1^Table 1 and Table 2 showed that age, professional title, hospital level, and safety of a COVID-19 vaccine were significant between two groups. A multiple logistic regression was used to analyze the relationship between these four variables with the acceptance of the COVID-19 vaccine.

**Table 4:** Reasons for acceptance of COVID-19 vaccination in 906 subjects.

Reasons	N (%)
Totally own decision, no specific reason	674 (74.4)
Hesitancy, but received following	212 (23.4)
Consulted the scientific literature	50 (5.5)
Encouraged by family members	7 (0.8)
Encouraged by collegues	23 (2.5)
Encouraged by friends	2 (0.2)
Encouraged by experts	17 (1.9)
Encouraged by news media	7 (0.8)
Requested by employer	106 (11.7)
Unwilling to, but followed employer’s mandate	20 (2.2)

**Table 5:** Reasons for decline of COVID-19 vaccination in 145 unvaccinated subjects.

Reason	N (%)
Totally own decision, no specific reason	6 (4.1)
Not willing to, but find other excuse, such as toothache, cold, and pregnancy preparation	5 (3.4)
Worry about the efficacy of the vaccine	1 (0.7)
Worry about short-term protection (3–6 months)	5 (3.4)
Influenced by collegues	2 (1.4)
Influenced by friends	0 (0)
Worry about political involvement	0 (0)
Worry about the safety of the vaccine without own chronic diseases	4 (2.8)
Worry about the safety because of own chronic diseases	36 (24.8)
Willing to take, but declined by vaccination staff	27 (18.6)
Pregnancy	19 (13.1)
Breastfeeding	19 (13.1)
Others	21 (14.5)
Pregnancy preparation	5 (3.4)
Acute respiratory infection	4 (2.8)
Allergic history	4 (2.8)
Injection of other vaccine	2 (1.4)
Vaccine not available	4 (2.8)
Toothache	2 (1.4)

## Data Availability

The data are available under reasonable request to the corresponding authors.

## Supplementary Materials

The following are available online at https://www.mdpi.com/article/10.3390/vaccines9070704/s1, Supplementary file 1: Questionnaire translated from Chinese to English. Table S1: Detailed adverse events in 312 vaccines after the first dose vaccine. Table S2: Detailed adverse events in 163 vaccines after the second dose vaccine.
