# Supplementary material for: Real-World Acceptance of COVID-19 Vaccines among Healthcare Workers in Perinatal Medicine in China
Source: Vaccines (Basel). 2021 Jun 27;9(7):704. doi: 10.3390/vaccines9070704 (PMC8310137; doi:10.3390/vaccines9070704)
Supplement: Supplementary file 1 [file vaccines-09-00704-s001.zip › vaccines-1263902-supplementary.pdf]

Questionnaire-Chinese and English

2021 太原-第十三届围产医学新进展高峰论坛
参会人员新冠疫苗接种调查表

Questionnaire on COVID-19 vaccination among participants in the 13th symposium in the progress of perinatal medicine in Taiyuan, China, 2021

本次调查的目的是为了解我国妇产科/围产医学以及密切相关的医务人员新冠疫苗的真实世界接种率以及相关问题。问卷是匿名的，是否回答该问卷，完全根据你自己的意愿，不会影响你的任何利益，也不会对你造成任何不良影响。如果你愿意参与本次调查，请你独立客观地回答各个问题，同时说明你知情同意参加本次调查。谢谢你的支持和合作！

This survey aims to investigate the real-world acceptance of COVID-19 vaccine and related issues among healthcare workers in obstetrics and gynecology, perinatal medicine, and closely related majors in China. The questionnaire is anonymous. Whether or not answering the questionnaire is all determined based on your own willingness, and will not influence any of your interests and will not cause any negative effect to you. If you are willing to participate in this survey, please complete the questionnaire independently and objectively. And meanwhile, it indicates that you have agreed to participate in the survey based on the informed consent. Thank you for your support and cooperation.

一、一般情况
I. Participants’ Characteristics

第 1 题 您所在省份城市与地区 [**填空题**]：____省____市____县
Q1: You are from [**Blank filling**]: _______ Province _______ City_______ County

第 2 题 民族 [**填空题**]：____。
Q2: Nationality [**Blank filling**]: _______.

第 3 题 宗教信息[**填空题**]：____ 。
Q3: Religion [**Blank filling**]:_______.

第 4 题 性别 [**单选题**]：男__ ；女__。
Q4: Gender [**Single choice**]: Male__; Female__.

第 5 题 您年龄段(岁) [**单选题**]： 18~30__；31~40__；41~50__；51~60__。
Q5: Your age (Year) [**Single choice**]: 18-30__; 31-40__; 41-50__; 51-60__.

第 6 题 最后学历 [**单选题**]：博士后__；博士__；硕士__；本科__；大专__；中专和中专以下__。
Q6: Your highest education [**Single choice**]: Postdoctoral researcher__; Doctor__; Master __; Undergraduate __; Junior college __; Technical secondary school and below __.

第 7 题 身份 [**单选题**]：医生__；护士/助产士__；技师/检验__；实验室研究人员__；行政人员__；其他 [注明具体身份]____________。
Q7: Your role in hospital [**Single choice**]: Doctor__; Nurse__; Midwife__; Technician__; Laboratory staff__; Administrative staff__; Other [Specify the details]______________.

第 8 题 科室名称 [**单选题**]：妇产科/生殖__；儿科__；其他[具体专业]__________。
Q8: Department [**Single choice**]：Gynecology/Obstetrics/Reproductive medicine __; Pediatrics__; Other [Specify the details]__________.

第 9 题 职称 [**单选题**]：正高__；副高__；中级__；初级__；其他[具体]_______。
Q9: Professional title [**Single choice**]: Senior__; Vice Senior__; Intermediate__; Junior__; Other [Specify the details]____________.

二、所属医疗机构
II. Employer Hospitals

第 10 题 医院等级 [**单选题**]三级__；二级__；一级__。
Q10: Hospital Level [**Single choice**]：Grade A__; Grade B__; Grade C__.

第 11 题 是否为大学附属医院 [**单选题**]是__；否__。
Q11: University hospital [**Single choice**]: Yes__; No__.

三、关于新冠疫苗的一般性知识
III. About the general knowledge of COVID-19 vaccine

第 12 题 我国目前接种最多的疫苗是哪一类 [单选题]
- 不知道__；
- 全病毒灭活疫苗__；
- 腺病毒重组疫苗__；
- 重组新冠蛋白亚单位疫苗__；
- 新冠 mRNA 疫苗__。

Q12. Currently, what kind of vaccine is the most vaccinated in China [Single choice]
- No idea__;
- Inactivated virus vaccines__;
- Adenovirus recombinant vaccine__;
- Recombinant coronavirus protein subunit vaccine__;
- Coronavirus mRNA vaccine__.

第 13 题 你新冠疫苗的知识来自于 [单选或多选]
- 各种新闻媒介__；
- 自己查阅专业期刊__；
- 听周围同事介绍__；
- 听专家介绍__；
- 参加单位组织的专门讲座__；

- 参加学术团体组织的学术会议__；
- 几乎不了解新冠疫苗__。

Q13. Your knowledge source about COVID-19 vaccine [Single or multiple choices]

- Various news media__;
- Professional journals__;
- Colleagues__;
- Experts lecture__;
- Lectures organized by your employer hospital__;
- Conferences organized by academic groups__;
- Little knowledge about coronavirus vaccine__.

第 14 题　你对疫苗的安全性 [单选题]

- 完全不担心/不担心__；
- 有些担心__；
- 担心/很担心__。

Q14. Your concern about the safety of COVID-19 vaccine [Single choice]

- Not worry at all/little worry__;
- Somewhat worry__;
- Worry/Very worry__.

第 15 题　你对疫苗的有效性 [单选题]

- 很有信心/有信心__；
- 比较有信心__；
- 有些信心__；
- 没有信心/完全没有信心__。

Q15. Your confidence about the efficacy of COVID-19 vaccine [Single choice]

- Very confidence/confidence__;
- Fair confidence__;
- Some confidence__;
- No confidence at all__.

四、新冠疫苗接种的真实世界情况

IV. Real-world situation about the COVID-19 vaccination

第 16 题　你是否接种了新冠疫苗 [单选题]

- 是__；
- 否__。

Q16. Did you receive the COVID-19 vaccine [Single choice]

- Yes__;
- No__.

➢ 如果你接种了新冠疫苗，请回答问题 17-26。如果你没有接种疫苗，请跳过问题 17-26，直接回答问题 27。

➢ If you received the COVID-19 vaccine, please answer the questions 17-26. If you did not receive the vaccine, please omit the questions 17-26, and answer question 27.

第 17 题　你接种新冠疫苗的理由是 [单选题]

- 完全是自己的决定，不需要理由__；
- 自己犹豫不决，查阅相应的科学文献后愿意接种__；
- 自己犹豫不决，受家庭成员(包括直系和表亲)的影响愿意接种__；
- 自己犹豫不决，受周围同事(不含专家或专业人员)影响愿意接种__；
- 自己犹豫不决，受自己的朋友或熟人(不含同事/专家/专业人员)的影响__；
- 自己犹豫不决，咨询专业人员或专家后愿意接种__；
- 自己犹豫不决，看有关疫苗新闻宣传后愿意接种__；
- 自己犹豫不决，工作单位规定要求接种__；
- 自己不愿意接种，工作单位规定要求接种，不得不接种__。

Q17. The reason for your acceptance of COVID-19 vaccination [Single choice]

- Based on own decision and no specific reason required__;
- Hesitant initially, but vaccinated after consulting the scientific literature__;
- Hesitant initially, but vaccinated after consulting family members (including immediate family members and cousins) __;
- Hesitant initially, but vaccinated after consulting colleagues (not including experts or professionals) __;
- Hesitant initially, but vaccinated after consulting friends or acquaintances (not including colleagues, experts or professionals) __;
- Hesitant initially, but vaccinated after consulting a professional or an expert__;
- Hesitant initially, but vaccinated after listen to news about COVID-19 vaccine__;
- Hesitant initially, but vaccinated after the request by the employer hospital__;
- Unwilling to get vaccinated, but had to be vaccinated at the request of the employer hospital__.

第 18 题　新冠疫苗需要间隔 2-4 周，共接种 2 针，你是否接种了 2 针疫苗 [单选题]

- 是__；
- 否，未接种第 2 针的原因是__________________________________。

Q18. The COVID-19 vaccination requires 2 doses at an interval of 2-4 weeks, did you receive the 2nd injection [Single choice]

- Yes__;
- No__; please specify the reason ________________________________.

第 19 题　接种第 1 针疫苗后是否出现不良反应 [单选题]

- 完全没有，包括没有局部疼痛、肿胀等__；
- 有__。

Q19. Are there any adverse reactions after the first dose of vaccine? [Single choice]

- No any adverse event, including local pain or swelling__;
- Yes __.

➢ 如果没有不良反应，请跳过问题 20-22，直接回答问题 23

➢ 如果有不良反应，请回答问题 20-22，然后继续回答问题 23

- If no any adverse event occurred, please omit the questions 20-22, and answer question 23.
- If any adverse occurred, please answer the questions 20-22 and then answer question 23.

第 20 题　第 1 针疫苗后具体不良反应是 [单选或多选]

- 局部反应__；
- 过敏反应__；
- 发热__；
- 流感样症状__；
- 头痛或头晕__；
- 失眠__；
- 消化系统症状__；
- 呼吸系统症状__；
- 月经失调__。

Q20. The detailed adverse events after the 1st vaccine dose [Single or multiple choices]

- Local reactions__;
- Anaphylactic reactions__;
- Fever__;
- Influenza-like symptoms__;
- Headache or dizziness __;
- Insomnia__;
- Digestive symptoms__;
- Respiratory symptoms__;
- Menstrual disorders__.

第 21 题：第 1 针疫苗后各种不良反应的细节

Q21. Detailed information of each adverse event after the 1st vaccine dose

A.　局部反应包括 [单选或多选]

- 疼痛__；
- 红肿__；
- 周围皮肤皮疹__；
- 周围皮肤溃烂__；
- 局部刺痛__；
- 影响肢体功能__；
- 其他__。

A. Local reactions [Single or multiple choices]:

- Pain__;
- Redness and swelling__;
- Surrounding skin rash__;
- Surrounding skin ulceration__;
- Local tingling__;
- Influence of limb function__;
- Others, please specify__.

B. 过敏反应为 [单选或多选]

- 皮疹__；
- 皮肤瘙痒__；
- 荨麻疹__；
- 喉头水肿__；
- 过敏性休克__。

B. Anaphylactic reactions [Single or multiple choices]

- Rash__;
- Pruritus__;
- Urticaria__;
- Larynx edema__;
- Anaphylactic shock__.

C. 发热程度 [单选题]

- 低热(<38 度) __；
- 中度发热(38~38.9 度) __；
- 高热(≥39 度) __。

C. Fever [Single choice]

- Low fever (<38°C) __;
- Moderate fever (38-38.9°C) __;
- High fever (≥39°C) __.

D. 流感样症状包括 [单选或多选]

- 乏力__；
- 全身酸痛__；
- 流涕或喷嚏或咽喉痛__；
- 咳嗽__。

D. Influenza-like symptoms [Single or multiple choices]

- Fatigue__;
- General pains and soreness__;
- Runny nose/sneezing/sore throat__;
- Cough__.

E. 头痛、头昏或眩晕 [单选或多选]

- 头痛__；
- 头昏__；
- 眩晕__。

E. Headache, dizziness or vertigo [Single or multiple choices]

- Headache__;
- Dizziness__;
- Vertigo__.

F. 消化系统症状 [单选或多选]

- 食欲减退__；
- 恶心__；
- 呕吐__；
- 腹痛__；
- 腹泻__。

F. Digestive symptoms [Single or multiple choices]

- Loss of appetite__;
- Nausea__;
- Vomiting__;
- Abdominal pain__;
- Diarrhea__.

G. 呼吸系统症状 [单选或多选]

- 咳嗽__；
- 咳痰__；
- 胸痛__；
- 呼吸急促__。

G. Respiratory symptoms [Single or multiple choices]

- Cough__;
- Sputum__;
- Chest pain__;
- Dyspnea__.

H. 月经失调 [单选或多选]

- 月经提前__；
- 月经延迟__；
- 出血期延长__；
- 出血期缩短__；
- 出血量增多__；
- 出血量减少__；
- 月经中期出血__。

H. Menstrual disorders [Single or multiple choices]

- Earlier menses__;
- Delayed menses__;
- Prolonged menstrual bleeding__;
- Shortened menstrual bleeding__;
- Heavy bleeding volume__;
- Light bleeding volume__;
- Intermenstrual bleeding__.

第 22 题 不良反应的严重程度 [单选题]

- 轻：不需要治疗__；
- 中：需要治疗，但不住院；用了哪些治疗(包括病休) __；
- 重：需要住院治疗；住院原因及天数__。

Q22. Severity of adverse events [Single choice]

- Mild: requiring no treatment__;
- Moderate: requiring treatment, including sick leave, but not hospitalization__;
- Severe: requirement of hospitalization__.

第 23 题 接种第 2 针疫苗后是否出现不良反应 [单选题]

- 完全没有，包括没有局部疼痛、肿胀等__；
- 有__。

Q23. Are there any adverse events after the second dose of vaccine [Single choice]

- No any adverse event, including local pain or swelling__;
- Yes __.

- ➢ 如果接种第 2 针疫苗后没有不良反应，问卷到此结束
- ➢ 如果接种第 2 针疫苗后有不良反应，请回答问题 24-26
- ➢ If no any adverse event after the second vaccine dose, stop answering question here.
- ➢ If any adverse occurred after the second vaccine dose, please answer the questions 24-26.

第 24 题 具体的不良反应是 [单选或多选]

- 局部反应__；
- 过敏反应__；
- 发热__；
- 流感样症状__；
- 头痛或头晕__；
- 失眠__；
- 消化系统症状__；
- 呼吸系统症状__；
- 月经失调__。

Q24. The detailed adverse events after the 1st vaccine dose [Single or multiple choices]

- Local reactions__;
- Anaphylactic reactions__;
- Fever__;
- Influenza-like symptoms__;
- Headache or dizziness __;
- Insomnia__;
- Digestive symptoms__;
- Respiratory symptoms__;
- Menstrual disorders__.

第 25 题 第 2 针疫苗后不良事件的细节

Q25. Detailed information of each adverse event after the 2nd vaccine dose

A. 局部反应包括 [单选或多选]

- 疼痛__；
- 红肿__；
- 周围皮肤皮疹__；
- 周围皮肤溃烂__；
- 局部刺痛__；
- 影响肢体功能__；
- 其他__。

A. Local reactions [Single or multiple choices]:

- Pain__;
- Redness and swelling__;
- Surrounding skin rash__;
- Surrounding skin ulceration__;
- Local tingling__;
- Influence of limb function__;
- Others, please specify__.

B. 过敏反应为 [单选或多选]

- 皮疹__；
- 皮肤瘙痒__；
- 荨麻疹__；
- 喉头水肿__；
- 过敏性休克__。

B. Anaphylactic reactions [Single or multiple choices]

- Rash__;
- Pruritus__;
- Urticaria__;
- Larynx edema__;
- Anaphylactic shock__.

C. 发热程度 [单选题]

- 低热(<38 度) __；
- 中度发热(38~38.9 度) __；
- 高热(≥39 度) __。

C. Fever [Single choice]

- Low fever (<38°C) __;
- Moderate fever (38-38.9°C) __;
- High fever (≥39°C) __.

D. 流感样症状包括 [单选或多选]

- 乏力__；

- 全身酸痛__；
- 流涕或喷嚏或咽喉痛__；
- 咳嗽__。

D. Influenza-like symptoms [Single or multiple choices]

- Fatigue__;
- General pains and soreness__;
- Runny nose/sneezing/sore throat__;
- Cough__.

E. 头痛、头昏或眩晕 [单选或多选]

- 头痛__；
- 头昏__；
- 眩晕__。

E. Headache, dizziness or vertigo [Single or multiple choices]

- Headache__;
- Dizziness__;
- Vertigo__.

F. 消化系统症状 [单选或多选]

- 食欲减退__；
- 恶心__；
- 呕吐__；
- 腹痛__；
- 腹泻__。

F. Digestive symptoms [Single or multiple choices]

- Loss of appetite__;
- Nausea__;
- Vomiting__;
- Abdominal pain__;
- Diarrhea__.

G. 呼吸系统症状 [单选或多选]

- 咳嗽__；
- 咳痰__；
- 胸痛__；
- 呼吸急促__。

G. Respiratory symptoms [Single or multiple choices]

- Cough__;
- Sputum__;
- Chest pain__;
- Dyspnea__.

H. 月经不调 [单选或多选]

- 月经提前__；
- 月经延迟__；
- 出血期延长__；
- 出血期缩短__；
- 出血量增多__；
- 出血量减少__；
- 月经中期出血__。

H. Menstrual disorders [Single or multiple choices]

- Earlier menses__;
- Delayed menses__;
- Prolonged menstrual bleeding__;
- Shortened menstrual bleeding__;
- Heavy bleeding volume__;
- Light bleeding volume__;
- Intermenstrual bleeding__.

第 26 题 不良反应的严重程度 [单选题]

- 轻：不需要治疗__；
- 中：需要治疗，但不住院；用了哪些治疗(包括病休) __；
- 重：需要住院治疗；住院原因及天数__。

Q26. Severity of adverse events [Single choice]

- Mild: requiring no treatment__;
- Moderate: requiring treatment, including sick leave, but not hospitalization__;
- Severe: requirement of hospitalization__.

第 27 题 没有接种新冠疫苗的原因是 [单选题]

- 自己不愿意接种，没有理由__；
- 自己不愿意接种，但找其他理由，如备孕、感冒、牙疼、工作太忙等借口没有接种__；
- 担心疫苗的效果不好，甚至无效__；
- 担心疫苗的效果维持时间仅 3-6 个月，长期效果不佳__；
- 担心疫苗从研究到应用，时间太短，还在观望__；
- 担心紧急批准的疫苗，疫苗与政治有关__；
- 受周围同事(不含专家或专业人员)影响而没有接种__；
- 受自己的朋友或熟人(不含同事，不含专家或专业人员)的影响而没有接种__；
- 受专家的影响而没有接种__；
- 自己身体健康，但担心疫苗的安全性__；
- 自己患有慢性病，担心疫苗的安全性，具体病种(可以多种) __；
- 自己想接种，但登记接种时被告知不能接种，不能接种的原因__；
- 其他(尽可能具体) __。

Q27. The reason for the decline of COVID-19 vaccine [Single choice]

- Unwilling to vaccinate for no reason__;
- Unwilling to vaccinate, but find other excuses, such as pregnancy preparation, catching cold, toothache, too busy to have time getting vaccinated, and others__;
- Worry about the efficacy of vaccine, or ineffective__;

- Worry about the short effective duration (only 3-6 months), and no long-term protection__;
- Worry about the short period from research to application and being wait-and-see__;
- Worry about the urgent approval due to political issue__;
- Influenced by colleagues (not including experts or professionals) __;
- Influenced by friends or acquaintances (not including colleagues, experts or professionals) __;
- Influenced by the experts or professionals__;
- Worry about the vaccine safety although in good health condition__;
- Worry about the vaccine safety because of the presence of chronic diseases, please specify the diseases__________________;
- Willing to be vaccinated, but declined by the vaccination staff, please specify the reasons__________________;
- Other reasons, please specify__________________.

Table S1. Detailed adverse events in 312 vaccinees after the first dose vaccine

Adverse events	n	Proportion (%)
Type of adverse events[a]		
Injection site (ache and/or swelling, no visible erosion)	231	74.0
Fever[b]	23	7.4
Flu-like illness	67	21.5
Headache and/or dizziness	94	30.1
Insomnia	13	4.2
Nausea and/or other digestive symptoms	20	6.4
Cough and/or other respiratory symptoms	10	3.2
Abnormal menstruation	7	2.2
Allergy	13	4.2
Severity of adverse events		
Very mild/Mild, no required treatment	291	93.3
Moderate, requirement of treatment, including sick leave	21	6.7
Severe, requirement of hospitalization	0	0

[a]Some of the 312 subjects had more than one adverse events. [b]<38°C, 20 subjects; 38-38.9°C, 3 subjects.

Table S2. Detailed adverse events in 163 vaccinees after the second dose vaccine

Adverse events	n	Proportion (%)
Type of adverse events[a]		
Injection site (ache and/or swelling, no visible erosion)	132	81.0
Fever[b]	5	3.1
Flu-like illness	19	11.7
Headache and/or dizziness	28	17.2
Insomnia	2	1.2
Nausea and/or other digestive symptoms	7	4.3
Cough and/or other respiratory symptoms	3	1.8
Abnormal menstruation	3	1.8
Allergy	5	3.1
Severity of adverse events		
Very mild/Mild, no required treatment	157	96.3
Moderate, requirement of treatment, including sick leave	6	3.7
Severe, requirement of hospitalization	0	0

[a]Some of the 163 subjects had more than one adverse events. [b]<38°C, 4 subjects; 38-38.9°C, 1 subject.
